# Exosomal microRNAs isolated from plasma of mesenteric veins linked to liver metastases in resected patients with colon cancer

**DOI:** 10.18632/oncotarget.16103

**Published:** 2017-03-10

**Authors:** Mariano Monzo, Sandra Santasusagna, Isabel Moreno, Francisco Martinez, Raquel Hernáez, Carmen Muñoz, Joan J. Castellano, Josep Moreno, Alfons Navarro

**Affiliations:** ^1^ Molecular Oncology and Embryology Laboratory, Human Anatomy Unit, School of Medicine, University of Barcelona, IDIBAPS, Barcelona, Spain; ^2^ Department of Medical Oncology and Surgery, Hospital Municipal de Badalona, Badalona, Spain

**Keywords:** exosomes, colon cancer, miR-328, microRNAs, tumor-draining vein

## Abstract

Before reaching a peripheral vein (PV), miRNAs released by the tumor are diluted and dispersed throughout the body or even retained in a specific organ. We hypothesized that blood drawn from the tumor-draining vein could provide more homogeneous information than blood drawn from the PV as that blood would contain all the biomarkers released by the tumor before they reach a potential metastatic site. We have profiled 754 miRNAs in 15 colon cancer plasma samples from the tumor-draining vein, the mesenteric vein (MV), identifying 13 microRNAs associated with relapse. The prognostic impact of these miRNAs were validated in 50 MV and 50 paired PV plasma samples of stage I-III colon cancer patients. Four miRNAs, let-7g, miR-15b, miR-155 and miR-328, were found overexpressed in MV compared to PV, and patients with high levels of those miRNAs in MV plasma had shorter time to relapse. Interestingly, in patients developing liver metastases, the exosomal cargo of miR-328 was much greater in MV than in PV plasma indicating a possible role of miR-328 in the development of liver metastases. Our results indicate that in colon cancer, the primary tumor releases high concentrations of miRNAs through the MV, and some of them are contained in tumor derived exosomes.

## INTRODUCTION

Colon cancer (CC) is the third most common cancer and the second cause of death from cancer in developed countries [1]. Disease stage is the primary prognostic factor for CC and surgery is the first therapeutic option for stages I-III - alone for stage I or followed by adjuvant chemotherapy in stage III. However, in patients with stage II disease, the benefit of adjuvant treatment is unclear [2]. While several studies have shown that oxaliplatin plus either 5-fluorouracil or capecitabine can prolong disease-free survival and overall survival by 20% in stage III patients [3, 4], the evidence in stage II patients is not so clearcut [5]. In recent years, the effect of the monoclonal antibodies cetuximab [6] and bevacizumab [7] has also been examined, but results have been less than satisfactory. There is thus a clear need for biomarkers that can predict relapse in patients with CC - particularly those with stage II disease - and thus help to identify patients likely to benefit from adjuvant therapy. Several circulating biomarkers have been examined in plasma or serum but findings are often inconsistent.

In recent years, microRNAs (miRNAs), non-coding RNAs that play a key role in the regulation of mRNA translation to protein, have emerged as promising biomarkers for screening, diagnosis and prognosis in several cancers, including CC. Several studies have found a link between miRNAs - either in tumor or in blood - and colorectal cancer. However, conflicting findings have been reported on the tumor expression levels of some miRNAs. For example, some studies have reported overexpression of miR-10, miR-23a, miR-34b, miR-150, miR-199b, miR-212, miR-296, miR-324, miR-331, miR-340, and let-7g in tumor samples, while others have reported underexpression of these miRNAs [8]. Moreover, differences in the expression level of the same miRNA are often observed between tumor and paired blood samples. For example, miR-92 was overexpressed in plasma [9] but underexpressed in tumor [10]. Similar inconsistencies were found in the expression of the miR-200 family, where high plasma expression of miR-200c was related to poor prognosis while high tumor expression of miR-200c was related to better prognosis [11].

More recent studies have focused on miRNAs associated with exosomes, small vesicles (30-100nm) that mediate cell-to-cell communication in a variety of biological processes. An exosome contains a small cytosol with both coding and non-coding RNAs, 40% of which are miRNAs [12]. Exosomes are secreted by several cell types and captured by receptor cells in many body fluids, where they regulate normal and pathological physiological processes. Exosomes play an active role in the metastatic process by modifying the surrounding stroma in order to prepare the tissue microenvironment for the anchoring of metastatic cells [13–15].

Studies analyzing the expression levels of exosomal miRNAs in blood samples report varying results, and no homogeneous miRNA profile for CC has been identified. For example, a recent study in serum samples found that the exosomal cargo of miR-17-92 cluster was associated with poor prognosis in metastatic patients with colorectal cancer [16], while another study in circulating exosomal miRNAs in metastatic colorectal cancer patients identified a set of completely different miRNAs that did not include the miR-17-92 cluster [17].

These inconsistent findings on miRNAs in tumor, biofluids, and exosomes in CC have been attributed to various causes, including different patient populations, internal controls, and laboratory methods [8]. However, one potential factor that has not been fully examined is the anatomic route that exosomes and their biomarker cargos take in order to colonize a target organ.

All studies examining biomarkers in blood have used blood samples drawn from a peripheral vein (PV) in the forearm. However, we hypothesize that this method limits the correct interpretation of results, since before reaching the forearm, potential biomarkers released by the tumor are diluted and dispersed in other parts of the body or even retained in a specific organ. For example, exosomal biomarkers released by a colon tumor may be retained in the liver and not continue their circulatory route to the forearm. In contrast, blood drawn from a mesenteric vein (MV) close to the tumor site may well contain all the biomarkers released by the tumor before they reach the potential metastatic site [18].

In order to test this hypothesis, we have profiled the miRNA expression in plasma samples taken from MV of surgically resected stage I-III CC patients and identified several MV-miRNAs associated with time to relapse (TTR). Furthermore, we confirm that these miRNAs were contained in MV-exosomes.

## RESULTS

### Patients

Fifty patients were included in the study. Mean age was 72 years, and 31 (62%) were males. Thirty-five patients had stage I-II disease. Of the 15 patients who developed metastases (5 stage II and 10 stage III), eight had liver metastases and seven had metastases in other organs. Mean follow-up was 45.2 months (range 26.4-63.8). K-ras mutations were assessed in 44 patients (Table 1).

### Identification of miRNAs associated to relapse

The analysis of 754 miRNAs in 15 MV samples allowed us to identify 13 miRNAs differentially expressed between relapsed *vs* non-relapsed patients. High MV expression of these miRNAs were associated with higher risk of relapse: let-7g (*p* = 0.008), miR-15b (*p* = 0.01), miR-18b (*p* = 0.04), miR-26a (*p* = 0.04), miR-106b (*p* = 0.04), miR-126 (*p* = 0.04), miR-142-3p (*p* = 0.04), miR-155 (*p* = 0.01), miR-328 (*p* = 0.02), miR-410 (*p* = 0.03), miR-449 (*p* = 0.03), miR-548c-5p (*p* = 0.04) and miR-744 (*p* = 0.02).

### Expression levels of relapse-associated miRNAs in MV and PV

**Figure 1:**
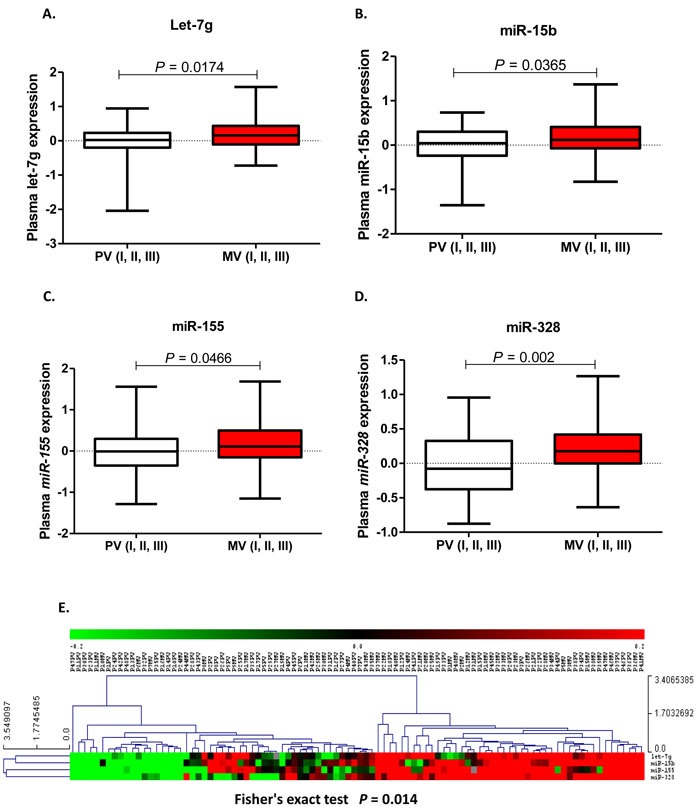
let-7g **A**., miR-15b **B**., miR-155 **C**. and miR-328 **D**. expression levels (fold change) in plasma from the peripheral vein (PV) and paired mesenteric vein (MV) of colon cancer patients. **E**. Heat map showing the miRNAs differentially expressed between PV and MV plasma samples clustered by Euclidean similarity metric (Fisher's exact *p =* 0.014). Each row represents one miRNA and each column represents a plasma sample. The legend on the right indicates the miRNA represented in the corresponding row. The relative miRNA expression is depicted according to the color scale. Red indicates upregulation; green indicates downregulation and gray indicates no detection. PV or MV indicates the region where the sample was obtained and the number indicates each patient.

Of the 13 relapse-associated miRNAs identified, four were expressed at higher levels in MV than in PV plasma: let-7g (*p =* 0.02); miR-15b (*p =* 0.04); miR-155 (*p =* 0.05); miR-328 (*p =* 0.002) (Figure 1A-1D). The Hierarchical cluster analysis of these four miRNAs confirmed that all four miRNAs obtained from MV had higher levels of expression than those obtained from PV (Fisher's exact *p =* 0.01) (Figure 1E). An association was observed between the MV expression levels of these four miRNAs and tumor site (let-7g: *p =* 0.03; miR-15b: *p =* 0.001; miR-155: *p =* 0.03; miR-328: *p =* 0.002) (Supplementary Figure 1). In addition, let-7g expression in MV was also associated with the presence of pre-existing polyps (*p =* 0.03), and miR-328 expression in MV was associated with disease stage (*p =* 0.07), and K-ras mutations (*p =* 0.04). There was no association between PV expression levels and clinical characteristics (Table 1).

**Table 1:** Patient characteristics and univariate p-values for time to relapse and for let-7g, miR-15b, miR-155 and miR-328 expression levels in 50 patients with early-stage colon cancer

			*P* value for association with microRNA expression							
			let-7g		miR-15b		miR-155		miR-328	
Characteristics	*N* (%), *N* =50	*P* value (TTR)	MV Plasma	PV Plasma	MV Plasma	PV Plasma	MV Plasma	PV Plasma	MV Plasma	PV Plasma
Sex		0.674	0.69	0.50	0.74	0.84	0.64	0.39	0.23	0.56
Male	31 (62)									
Female	19 (38)									
Median age	72	0.921	0.81	0.36	0.43	0.46	0.67	0.35	0.77	0.52
CEA levels		0.401	0.37	0.81	0.97	0.80	0.93	0.77	0.81	0.72
<=5	34 (68)									
>5	16 (32)									
C 19.9 levels		0.478	0.82	0.69	0.39	0.82	0.74	0.38	0.43	0.32
<=37	46 (92)									
>37	4 (8)									
Tumor location*		0.557	**0.03**	0.77	**0.001**	0.97	**0.03**	0.53	**0.002**	0.52
Left colon	26 (52)									
Right colon	24 (48)									
Tumor size (cm)		0.285	0.86	0.43	0.84	0.84	0.44	0.13	0.82	0.21
<=5	37 (74)									
>5	13 (26)									
Histological type		0.672	0.61	0.57	0.39	0.42	0.55	0.98	0.82	0.96
Well differentiated	45 (90)									
Poorly differentiated	5 (10)									
Pre-existent polyp		0.666	**0.03**	0.77	0.74	0.95	0.41	0.26	0.16	0.1
Absent	38 (76)									
Present	12 (24)									
Perilymphatic invasion		0.710	0.62	0.50	0.29	0.01	0.82	0.51	0.31	0.3
Absent	47 (94)									
Present	2 (4)									
Unknown	1 (2)									
Adjuvant treatment		0.405								
Fluoropyrimidines	28 (56)									
None	22 (44)									
TNM stage		0.001	0.16	0.72	0.26	0.31	0.62	0.97	**0.07**	0.33
I-II	35 (70)									
III	15 (30)									
Lymph nodes examined <12 >12	11(22) 39(78)	0.266								
K-ras mutations		0.79	0.11	0.45	0.65	0.43	0.32	0.78	**0.04**	0.39
Yes	14 (28)									
No	30 (60)									
Not assessed	6 (12)									
Relapsed			0.86	0.09	0.57	0.38	0.54	0.31	0.48	0.11
Yes	15 (30)									
Hepatic metastasis			0.83	0.25	0.45	0.78	0.61	0.58	0.71	0.12
Yes	8 (16)									

Abbreviations: CEA, Carcinoma Embryonic Antigen; MV, mesenteric vein; PV, peripheral vein; TNM, Tumor, Nodule, Metastasis; TTR, time to relapse.

*Tumor location according to anatomical site: 17 ascending colon, 7 transverse colon, 6 descending colon and 20 sigmoid colon.

### Plasma miRNA expression and TTR

**Figure 2:**
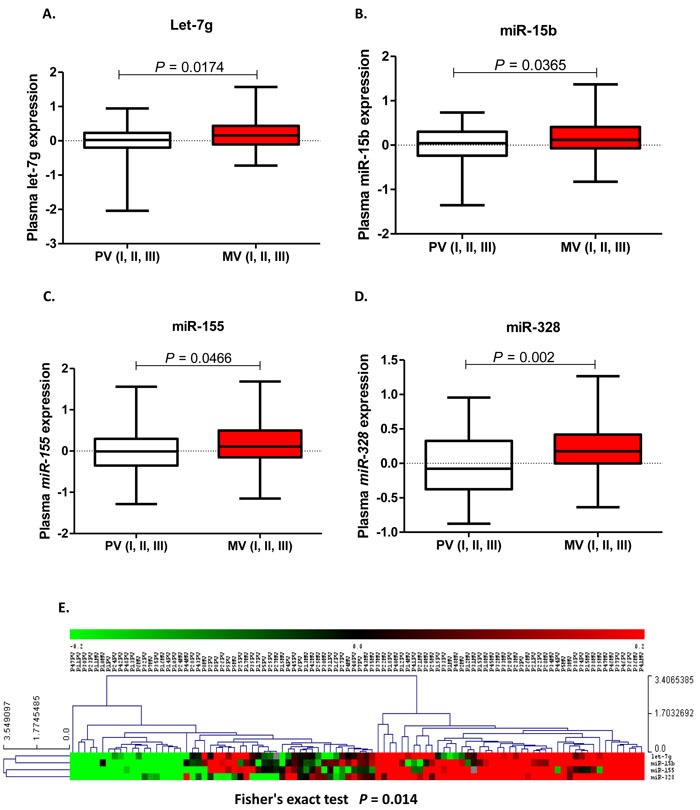
TTR according to let-7g **A**., miR-15b **B**., miR-155 **C**. and miR-328 **D**. expression levels (fold change) in plasma from the mesenteric vein (MV).

High MV expression of let-7g, miR-15b, miR-155 and miR-328 was associated with shorter TTR. Mean TTR was not reached for patients with low or high expression of let-7g (*p =* 0.05). TTR was 58.9 months (95% CI 52.6-65.3 months) for those with low levels of miR-15b, compared to 38.7 months (95% CI 29.9-47.6 months) for those with high levels (*p =* 0.004). TTR was 59.4 months (95% CI 53.5-65.2 months) for those with low levels of miR-155 and 41.1 months (95% CI 32.3-49.7 months) for those with high levels (*p =* 0.01). TTR was 55.7 months (95% CI 49.3-62.1 months) for patients with low miR-328 expression, compared to 35.1 months (95% CI 26.1-44.1 months) for those with high expression (*p =* 0.02) (Figure 2).

**Figure 3:**
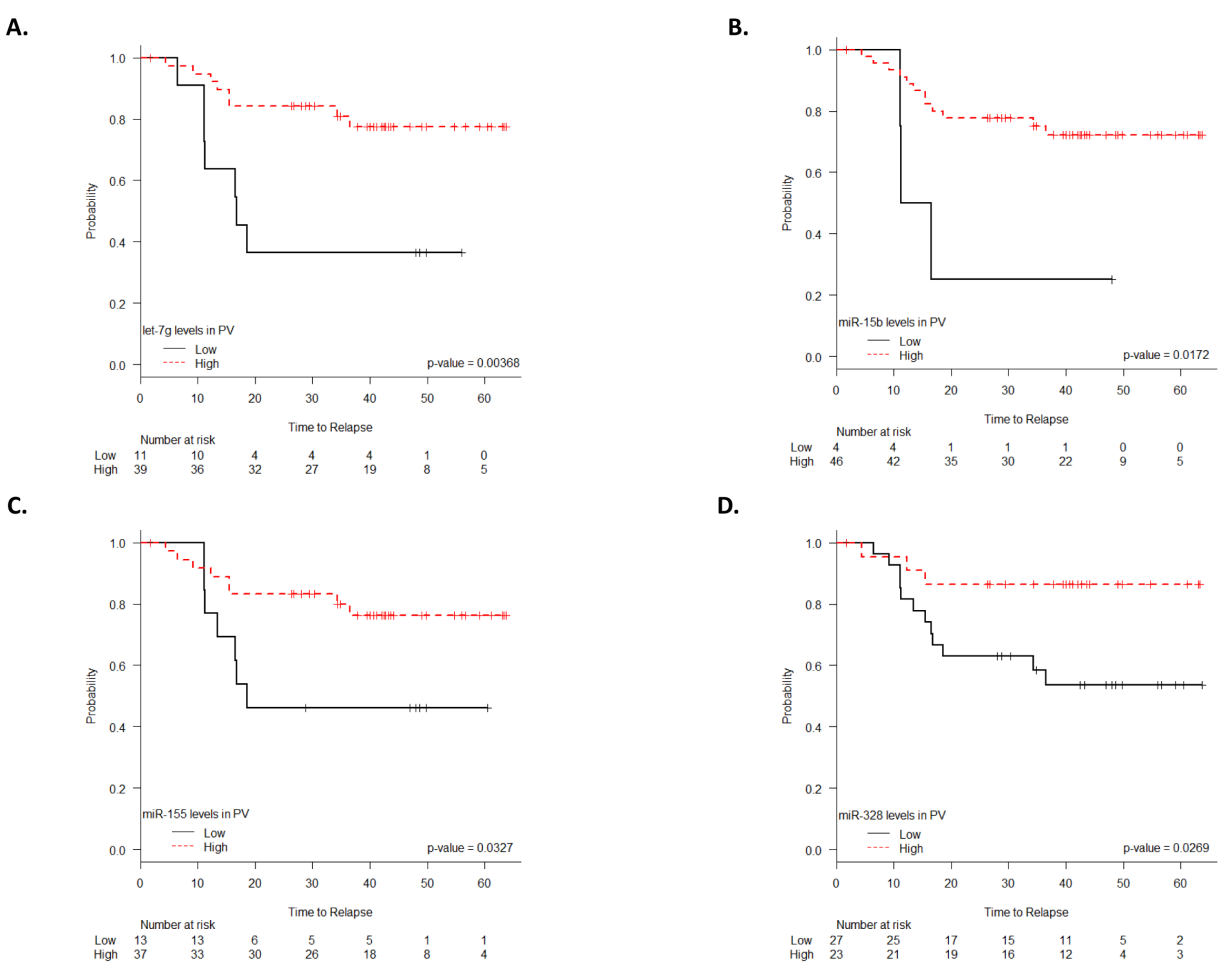
TTR according to let-7g **A**., miR-15b **B**., miR-155 **C**. and miR-328 **D**. expression levels (fold change) in plasma from the peripheral vein (PV).

In contrast, inverse results were found when PV was analyzed. High PV expression of these four miRNAs was associated with longer TTR. Mean TTR for patients with low let-7g expression was 28.7 months (95% CI 16.4-41.1 months), while it was 53.6 months (95% CI 47.3-59.9 months) for those with high expression (*p =* 0.004). TTR was 21.7 months (95% CI 6.7-36.7 months) for those with low levels of miR-15b, compared to 50.7 months (95% CI 44.3-57.1 months) for those with high levels (*p =* 0.02). TTR was 35.5 months (95% CI 29.9-48.1 months) for those with low levels of miR-155 and 52.9 months (95% CI 46.1-59.6 months) for those with high levels (*p =* 0.03). TTR was 42.3 months (95% CI 33.1-51.4 months) for patients with low miR-328 expression, compared to 56.1 months (95% CI 48.5-63.7 months) for those with high expression (*p =* 0.03) (Figure 3).

The multivariate analysis identified stage (HR = 0.08, 95% CI 0.02-0.32; *P <* 0.001) and high miR-328 expression in MV plasma (HR = 6.034, 95% CI 1.456-25; *p =* 0.01) as independent prognostic markers of TTR.

### miRNA cargo in exosomes isolated from MV and PV plasma

**Figure 4:**
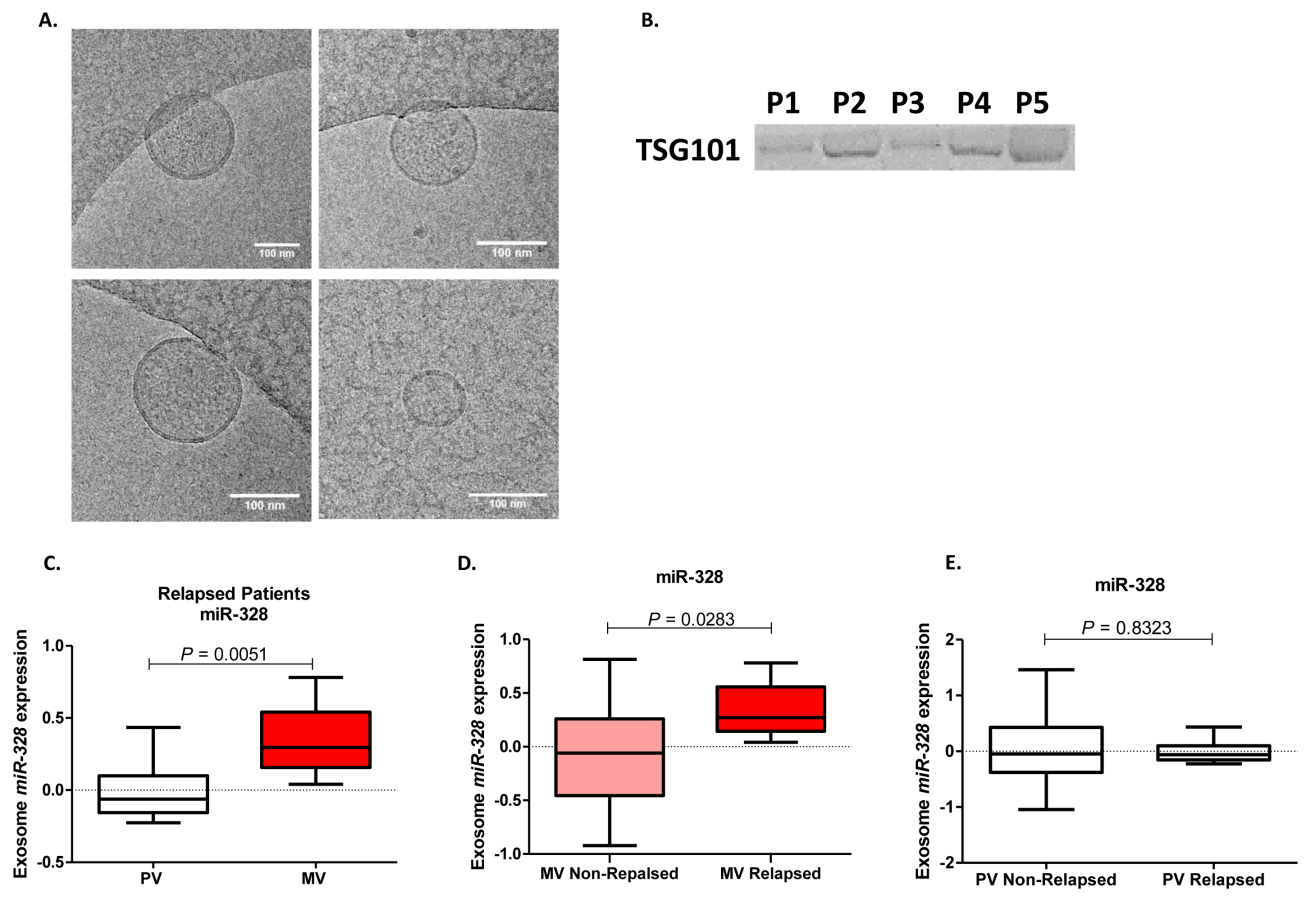
Exosome characterization in plasma samples of colon cancer patients by Cryo-TEM A. and Western blot using TSG101 marker B **C**. miR-328 expression levels (fold change) in exosomes from the peripheral vein (PV) and matched mesenteric vein (MV) of eight patients who developed liver metastases. **D**. miR-328 expression levels (fold change) in exosomes from the MV of patients with liver metastases compared to those without metastases. **E**. miR-328 expression levels (fold change) in exosomes from the PV of patients with liver metastases compared to those without metastases.

Sufficient plasma from both MV and PV to perform exosome isolation was only available from 33 patients (25 metastasis-free and eight with liver metastases). We captured images of round microvesicles with 30-100nm diameters in the exosome-rich fraction. Cryo-TEM and Western blot analyses confirmed the presence of exosomes in plasma samples (Figure 4A-4B).

In the samples from all 33 patients, there were no differences in the exosome cargo of let-7g, miR-15b, miR-155 and miR-328 in MV *versus* PV plasma (Supplementary Figure 2). In order to examine whether the exosome cargo of these miRNAs was greater in patients with liver metastases, we then analyzed the subset of eight patients with metastases and found that the miR-328 cargo in exosomes was greater in MV than in PV (*p =* 0.005) (Figure 4C). Moreover, when we compared exosomes isolated from MV from patients with liver metastases and those without, the miR-328 cargo was higher in metastatic patients (*p =* 0.03) (Figure 4D), indicating that miR-328 is expressed at low levels in patients without liver metastases. In exosomes isolated from PV, there was no difference between the two groups of patients (Figure 4E).

## DISCUSSION

Metastases are the primary cause of death in cancer patients. Recent studies have found that the primary tumor can release tumor-associated molecules into blood circulation, leading to the development of metastases in target organs [19]. Many investigators have examined the presence of miRNAs or exosomal miRNAs in plasma or serum samples from cancer patients [8, 11, 16, 17]. Since all biomarkers released by the primary tumor are released *via* veins, it is logical to suppose that more biomarkers would be located in veins close to the primary tumor. The large intestine is an optimal model to study exosome circulation due to its distinctive distribution of arteries and veins. Venous return of the colon occurs through the superior and inferior MVs, both of which flow into the hepatic portal vein, which carries blood to the liver. This anatomic distribution may explain the higher frequency of liver metastases associated with primary tumors located in the colon. For this reason, we have focused our study on patients with liver metastases. Furthermore, we have not included patients with rectal cancer, since the venous return of the rectum occurs through the iliac veins and does not flow into the liver.

**Figure 5:**
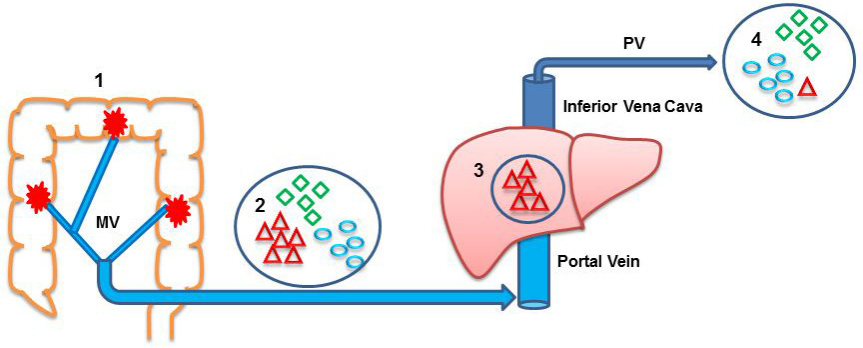
Anatomic route of miRNAs released by the primary colon tumor Venous return of the colon occurs through the mesenteric veins (MV). Both MVs flow into the hepatic portal vein, which carries blood to the liver through the inferior cava vein before continuing to the peripheral veins (PVs). This anatomic distribution may explain the higher frequency of liver metastases associated with primary tumors located in the colon. 1, colon; 2, miRNA content in MV; 3, liver; 4, miRNA content in PV. Triangles indicate miRNAs with metastatic potential; ovals and diamonds indicate miRNAs without metastatic potential.

In the present study, we have found that four miRNAs (let-7g, miR-15b, miR-155, miR-328) are more highly expressed in MV plasma than in PV plasma. Moreover, although all four miRNAs are associated with TTR, their overexpression in MV is related to poor prognosis, while their overexpression in PV is related to good prognosis. We can speculate that miRNAs with greater metastatic potential may be retained in the target organ, such as the liver, while those with less metastatic potential may continue to circulate towards the PV. Therefore, miRNAs isolated from PV blood would be more diluted than those from MV blood, have less metastatic potential, and thus indicate better prognosis (Figure 5). Moreover, the multivariate analysis identified overexpression of one of the miRNAs - miR-328 - in MV as an independent prognostic factor conferring a six-fold greater risk of relapse. Along these lines, a recent study in resected non-small-cell lung cancer patients found that tumor cells detected in the tumor-draining pulmonary vein were associated with relapse, while no association was found for tumor cells in the PV [20].

It is well known that exosomes and their cargo are one of the most important mediators of crosstalk between tumor cells and the microenvironment [21]. The presence of exosomes in blood has been analyzed in several tumors, including melanoma [22] and colorectal cancer [16, 17], where blood samples were taken from the PV of metastatic patients. Another study in metastatic ovarian cancer assessed exosomes in ascitic liquid [23]. All these studies found greater numbers of exosomes in plasma from patients than from healthy controls [16, 17].

Interestingly, our study included only I-III stage CC patients who had not developed metastases at the time of entering the study. It would thus be logical to expect that in these stages, the exosome cargo of our four miRNAs (let-7g, miR-15b, miR-155 and miR-328), would be greater in MV than in PV. However, when we analyzed both relapsed and non-relapsed patients, we found no overall significant differences in the miRNA cargo between exosomes isolated from MV and PV plasma. These findings are in line with those of previous studies [11, 24, 25], where higher expression levels of miRNAs in PV blood of metastatic patients was the manifestation of all miRNAs released by both the primary tumor and the metastasis.

In contrast, when we focused on the subset of patients who relapsed and developed liver metastases, while differences were still not found for let-7g, miR-15b or miR-155, the miR-328 exosomal cargo was much greater in MV than in PV. In addition, when the exosomal cargo in MV plasma in patients who developed liver metastases was compared to that in non-metastatic patients, the miR-328 cargo was higher in those with metastases. These findings lead us to suggest that the presence of exosomes with miR-328 cargo in MV plasma may well be an important predictor of metastases in CC patients. Along these lines, overexpression of miR-328 was related to brain metastases in patients with non-small-cell lung cancer [26].

There were more associations between miRNA expression in MV than in PV and clinical characteristics. In MV - but not PV - plasma, all four miRNAs were associated with tumor site, let-7g was associated with pre-existent polyp, and miR-328 was associated with disease stage. These findings indicate that biomarkers isolated from tumor-draining veins in patients with CC may provide much more clinical information than those from PVs.

Clearly, exosomes can help disseminate tumors and develop metastases intraperitoneally *via* other anatomic routes, including the lymphatic system. Tumors can also shed cells directly into the peritoneal cavity or the intestinal wall can be accidentally perforated during surgery [23, 27]. Our findings show that obtaining biomarkers from MV blood is a simple surgical method that can complement clinical findings.

In summary, our findings indicate that expression levels of let-7g, miR-15b, miR-155 and miR-328 isolated from MV blood can be a useful tool to identify patients at higher risk of relapse and that the miR-328 exosome cargo in MV blood may play an important role in modulating the tissue microenvironment for the anchoring of metastatic cells. Our results provide the first step towards obtaining more homogeneous results in the analysis of blood biomarkers and can constitute an excellent surgical method for identifying patients with CC who are at risk of developing liver metastases.

## MATERIALS AND METHODS

### Patients

From August 2009 to August 2013, samples were obtained from 50 patients with stage I-III CC who underwent surgical resection at the Municipal Hospital of Badalona (Badalona, Spain). Fifteen patients later relapsed, eight of whom had liver metastases (Table 1). All 50 patients had undergone a complete history and physical examination prior to surgery. Approval for the study was obtained from the institutional review board of the hospital, and signed informed consent was obtained from all patients in accordance with the Declaration of Helsinki.

### Blood samples

For all 50 patients, we obtained paired MV and PV blood as previously described [18]. On the day of surgery, 5 mL of blood was drawn from the PV and stored in heparinized tubes. During surgery, with vascular ligation before tumor resection, an additional 5 mL of blood was drawn from either the superior or the inferior MV, according to the anatomic location of the tumor.

### Sample processing and total RNA extraction

Plasma from all blood samples was obtained by centrifugation of the whole blood at 5000G during 10 min and saved frozen at -80°C until further use.

Total RNA was isolated from 250μl of plasma using miRNeasy Mini Kit (Qiagen, Valencia, CA, USA) according to the manufacturer's protocol. The concentration of total RNA was quantified using NanoDrop ND-1000 spectrophotometer (Thermo Scientific, Wilmington, DE, USA).

Supplementary Figure 3 shows the successive stages of the miRNA analyses performed.

### miRNA profiling

miRNA profiling of 754 different human miRNAs was performed using TaqMan Array Human microRNA Set Cards v3.0 (Applied Biosystems, Foster City, CA, USA) in a training set of 15 MV and 3 PV plasma samples as previously described [28]. Briefly, RT reactions of 4.50μl contained: 0.80μl of 10X RT buffer (Applied Biosystems), 0.2μl dNTPs (100mM each), 1.5μl multiscribe reverse transcriptase (50 U/μl), 0.10μl RNase inhibitor (20 U/μl), 0.80μl Megaplex RT primers (10X), 0.90μl of MgCl_2_ (25 U/μl) and 70ng of total RNA. RT reactions were incubated in a 2720 thermocycler (Applied Biosystems) for 2 min at 16°C and 1 min at 42°C for 40 cycles, 1 s at 50°C and 5 min at 85°C, and then held at 4°C. 2.5 μL of each RT product was preamplificated in order to increase the quantity of desired cDNA. Preamplification reactions were incubated in a 2720 thermocycler (Applied Biosystems) for 10 min at 95°C, 2 min at 55°C and 2 min at 72°C; then 15 s at 95°C and 4 min at 60°C for 12 cycles, 10 min at 99.9°C and then held at 4°C. Preamplification product was diluted with 75μL of 0.1X TE buffer (pH 8.0). Quantitative real-time PCR reactions were performed on an ABI 7500 HT Sequence Detection System (Applied Biosystems) and contained 9μL of the diluted preamplificated product, 450μL of TaqMan Universal PCR Master Mix, No AmpErase UNG 2X (Applied Biosystems) and 441μL of nuclease-free water, mixed by vortexing, and pipetted into microfluidic TaqMan Human miRNA Array A and B.

### Filtering and normalization of miRNA data

All miRNAs that were not expressed in all 15 MV plasma samples or were expressed with an unreliable quantification (Ct > 37) were excluded from further analysis, leaving a working set of 249 miRNAs. Relative miRNA expression was calculated using the 2 ^−∆∆Ct^ method. Normalization was performed with miR-484 as this miRNA was found to be the most stably expressed in all the samples.

### Identification of miRNAs associated with relapse

The potential association between the 249 miRNAs and relapse was analyzed with the class comparison tool from BRB Array tools. A random-variance *t*-test [29] was used to detect miRNAs that were differentially expressed in relapsed *vs* non-relapsed patients.

### Validation of miRNAs in the entire cohort

The miRNAs related to relapse were then used to evaluate TTR in the entire cohort of 50 patients using individual TaqMan microRNA assays (Applied Biosystems) in the ABIPrism 7500 Sequence Detection System (Applied Biosystems) as previously described [28].

### Exosome isolation, characterization and miRNA cargo

Exosome isolation was performed from 250μL of plasma samples by ultracentrifugation as previously described [30]. Briefly, sequential centrifugation at 4°C at 300G 5 min, followed by 2,500G 20 min and finally, 10,000G 30 min, followed by ultracentrifugation at 100,000G 2 hours. Then the pellet was washed with DPBS and ultracentrifuged again at 100,000G 1 hour in a Sorvall MX Plus Micro-Ultracentrifuge with S140AT Rotor and Policarbonate Tubes (Thermo Scientific).

Exosome characterization was done by two methods: 1) Cryo transmission electron microscopy (cryo-TEM) in a Jeol JEM 2011 transmission electron microscope at the Microscope Facility of the Autonomous University of Barcelona; and 2) western blot analysis, using the exosome marker TSG101.

For the analysis of exosome miRNA cargo, RNA was purified using miRNeasy Mini Kit (Qiagen) after resuspension of the exosome pellet obtained by ultracentrifugation in 750μl Qiazol. MiRNA expression was analyzed using single TaqMan miRNA assays.

### Statistical analyses

All 50 patients were evaluable for TTR, calculated from the date of surgery to the date of relapse or last follow-up. The univariate analysis of TTR according to miRNA expression was performed with the Kaplan-Meier method and compared using the log-rank test. Only covariates significantly associated with outcomes in the univariate analysis (two-sided *P* value < 0.05) were included in the Cox multivariate regression model. Results were reported as hazard ratios (HR) with their 95% confidence intervals (CI). To identify differences in expression according to two or more characteristics, including the comparison between miRNA levels in MV *vs* PV, the Students *T*-Test or ANOVA were used as appropriate.

All statistical analyses were performed with SPSS 22 (SPSS Inc, Chicago, IL), R 2.6.0 Software (Vienna, AU), BRB Array Tools version 3.5.0 software (Richard Simon & BRB-ArrayTools Development Team, http://linus.nci.nih.gov/BRB-ArrayTools.html, National Cancer Institute, Bethesda, MD, USA) and TIGR Multiexperiment viewer version 4.0 software (Dana-Farber Cancer Institute, Boston, MA). Statistical significance was set at *P* < 0.05.

## SUPPLEMENTARY MATERIALS FIGURES


