# Supplementary material for: Exosomal microRNAs isolated from plasma of mesenteric veins linked to liver metastases in resected patients with colon cancer
Source: Oncotarget. 2017 Mar 10;8(19):30859–69. doi: 10.18632/oncotarget.16103 (PMC5458173; doi:10.18632/oncotarget.16103)
Supplement: Supplementary file 1 [file oncotarget-08-30859-s001.pdf]

Exosomal microRNAs isolated from plasma of mesenteric veins linked to liver metastases in resected patients with colon cancer

Supplementary Material

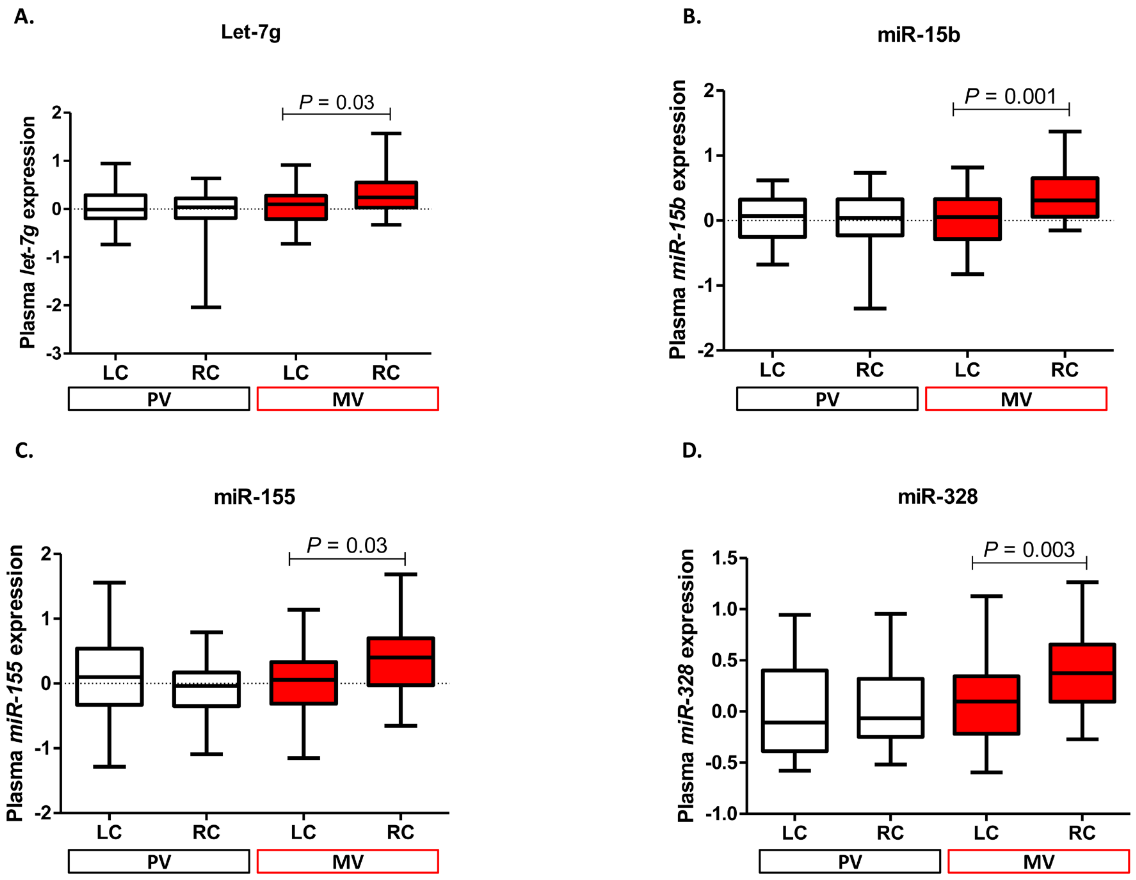

LC, left colon; RC, right colon.

Supplementary Figure 1: let-7g (A), miR-15b (B), miR-155 (C) and miR-328 (D) expression levels (fold change) in plasma from the peripheral vein (PV) and matched mesenteric vein (MV) of colon cancer patients classified by the anatomic location of the tumor.

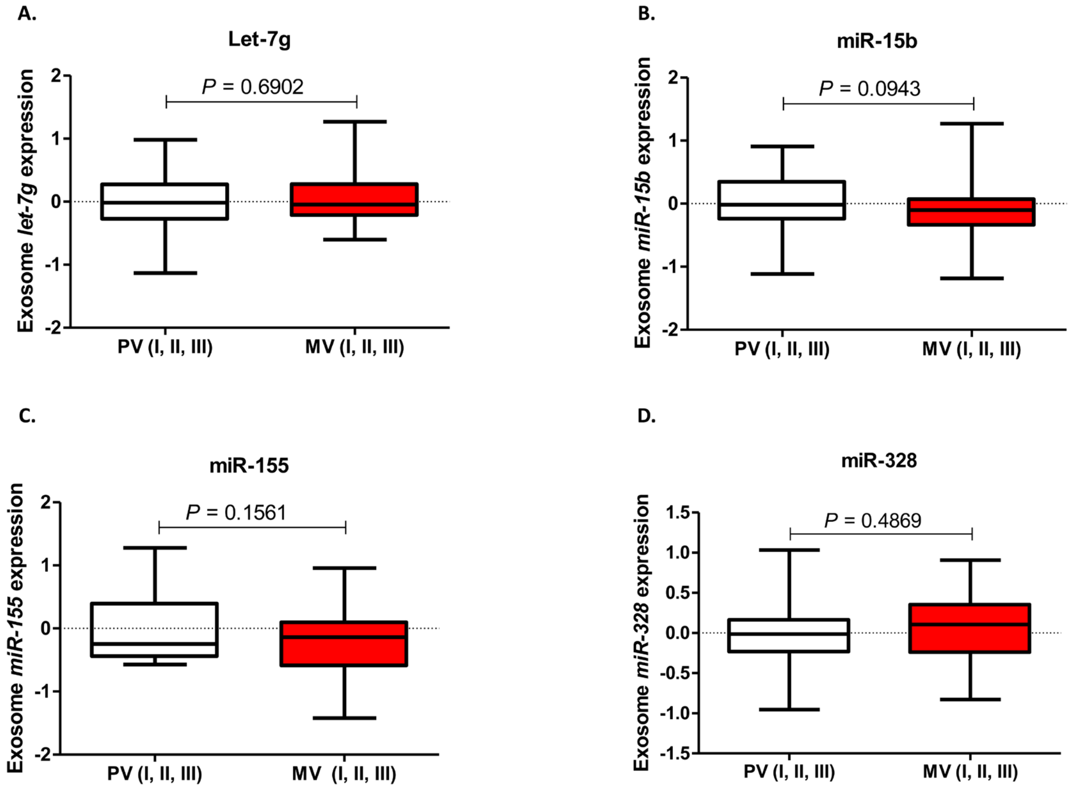

Supplementary Figure 2: let-7g (A), miR-15b (B), miR-155 (C) and miR-328 (D) expression levels in exosomes isolated in plasma from the peripheral vein (PV) and matched mesenteric vein (MV) of colon cancer patients.

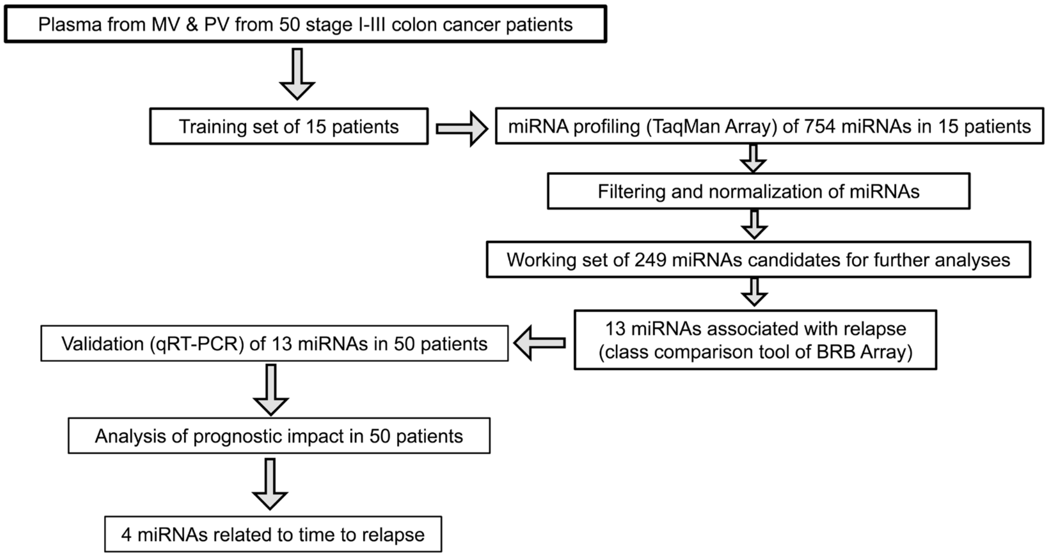

MV, mesenteric vein; PV, peripheral vein

Supplementary Figure 3: Stages of analyses for the identification of specific exosomal miRNAs associated with relapse in stage I-III colon cancer patients.
